# Hospital Festivals and Meetings

**Published:** 1894-06-02

**Authors:** 


### HOSPITAL FESTIVALS AND MEETINGS.

### HOSPITAL FOR SICK CHILDREN, GREAT ORMOND STREET.

The annual meeting took place in the board room of this hospital on May 23rd, Mr. A. Lucas presiding. The report presented to the Governors is not financially a cheering one, the institution being unfortunately very largely in debt, so much so, in fact, that the committee will be under the necessity of closing some of the wards unless some improvement is made in the financial condition of the institution. On the building fund alone the debt amounts to £9,350, the new wing having been opened last year on June 24th by the Prince and Princess of Wales. There would be no more building necessary, the Chairman said, except in the case of the north wing, where the wards for infectious diseases would be extended. The expense of this undertaking would be borne entirely by the Misses Cohen, who had chosen this way of raising a memorial to the late Countess of Rosebery.

Mr. Justice Kekewich moved that the chairman should be requested to communicate to Sir Henry Ponsonby the thanks of the committee for the gift of £100 from Her Majesty the Queen, and likewise to express their thanks to the Duke of York for presiding at the festival dinner of the hospital.

With regard to this festival dinner, the Chairman remarked that though the sum collected on the occasion—£10,000—might seem large, it was only really sufficient to enable them to keep on to the end of the year without any reduction of the work of the institution.

Votes of thanks to the officials of the hospital and to the chairman were passed before the conclusion of the meeting.

### EAST LONDON HOSPITAL FOR CHILDREN.

The annual general meeting was held at the above hospital on May 24th, the chair being taken by Mr. H. W. Trinder. The report presented to the Governors chronicled a large increase, about 25 per cent., in the number both of in-patients and out-patients, an increase due largely to the accommodation given by the new out-patient department, but also, in some measure, to the bad times and consequent underfeeding in the East-end. The increased expenses necessitated by the extension of the work of the hospital have not, however, been met by a corresponding increase of receipts, and had it not been for an opportune legacy, the board had feared, towards the end of 1893, that they would be obliged to restrict the work of the institution. The Chairman, in his introductory address, said that he thought, on the whole, they might look on the past year as a very successful one. They had been able to extend their work among the suffering poor, in spite of many difficulties, and although they had at one time been afraid that their endeavours would be crippled for lack of funds, yet thanks to legacies and to a gift of £5,000 from Mr. Charles Prescott, they had not only tided over their immediate difficulties but had been enabled to somewhat increase their invested property. He also appealed for help for the convalescent home to be established at Clacton-on-Sea, and for which a site had already been secured. In view of the establishment of this home certain alterations in the constitution of the hospital were made with the consent of the meeting; and with the usual votes of thanks the proceedings came to an end.

### GROSVENOR HOSPITAL FOR WOMEN AND CHILDREN.

The annual meeting held at this hospital on May 24th was very well attended, among those present being Lord Llangattock (chairman), Lady Llangattock, and the Hon. Miss Rolls, Lady Mordaunt, Mr. A. T. Ram (treasurer) and the Hon. Mrs. Ram, Mr. Justice Grantham, the Rev. George Napier, Mr. F. A. Bosanquet, Q.C., Mr. Beamish, and Mr. F. W. Fane. The Treasurer, in moving the adoption of the report, said that owing to legacies during the last year the income of the institution had exceeded its expenditure by the sum of about £18, but they must remember that they were in a chronic state of debt, and that in order to set the finances of the hospital on a satisfactory footing an increase of £200 a year on their present income was necessary. They wanted more annual subscribers. He thought that the institution suffered from not being well enough known, by reason of its being situated in so quiet a part of the town, a state of things, however, which had its advantages for the patients. He would also urge the need of augmenting the building fund, for which £6,000 or £8,000 was required. They had no wish to build a large hospital, but what they wanted was a hospital built for the purpose and not, as at present, two or three houses not originally intended for the reception of the sick. The Chairman, who followed, said that he thought they had much to be grateful for to their unpaid medical staff, and that he hoped some day they would be able to extend the work done amongst children, at present confined to the out-patient department. Mr. Bosanquet, the next speaker, read an extract from The Hospital of September 23rd, 1893, which, while giving the cost of management of their institution at about 10½ per cent., advised the committee to increase the emolument of the secretariat, and to spend more in raising money. With regard to the first part of the recommendation he could only say it was at present unfortunately impossible; with regard to the second, he did not know how it was to be done. In fact the only charge against them seemed to be that they were too economical. Speeches were also made by Mr. Godfrey Walter, Mr. Butler-Symthe, the Rev. G. Napier, and Mr. Justice Grantham, who moved the concluding vote of thanks to the chair.

### ST. MARY'S MATERNITY CHARITY, PLAISTOW.

The first annual meeting of the St. Mary's Maternity Charity and District Nurses' Home, Plaistow, was held at the Mansion House on Wednesday, May 25th, Lord Winchilsea, in the absence of the Duke of Westminster, taking the chair. Amongst others on the platform were Lord Robert Cecil, the Dean of St. Paul's, the Hon. Sydney Holland and General Gordon Pritchard. Lord Winchilsea first spoke of the good work now being done through the exertions of Sister Katherine (Miss K. Twining), who being deeply impressed with the terrible want of skilled maternity nursing amongst the poor, originated the Charity as one connected with the parish of St. Mary's, Plaistow in 1889. By the year 1892 its sphere had so widened that re-organization upon a larger basis was felt to be necessary, and it is now proposed to send a memorial to the Queen, asking for her Majesty's special patronage, and the permission to change the name of the institution to that of the Royal Maternity Nursing Home. The Central Home at Plaistow occupies four houses, and there are also branches at Custom House and Canning Town, some 35 nurses now being employed. During the past year more than 30,000 visits were altogether paid by the nurses, and the work apparently increases day by day. This part of the

Charity's work is of course entirely admirable, but a difficult question comes to the front with the introduction of a scheme of district nursing, which its promoters are proposing to carry on in country villages. Lord Winchilsea explained that in small country parishes great difficulty is experienced in finding the requisite funds to properly pay a thoroughly trained nurse, and that it is therefore suggested to send up certain selected women from the country villages, and to give them six months' training under Sister Katherine's supervision. They will then return to their native district, there to take up "any class of country cases," "under proper inspection." They hoped to become affiliated to the Queen's Jubilee Institute, the nurses of the latter association undertaking the necessary supervision. The last is a saving clause, but the provision of such inspection will be, we fear, somewhat hard of attainment. The Chairman said they were specially anxious not to flood the country with half-trained women, but it is a question how this is to be guarded against, if women with six months' training are to take up a position as "nurses" among a more or less ignorant country population, who will fail to grasp the difference between "general" and special qualifications for the work. The Hon. Sydney Holland, in an amusing speech, testified warmly to the good work being carried on in the East-end under great difficulties and drawbacks, and in most inadequate quarters, by Sister Katherine and her energetic staff, appealing to those present to help her by contributing towards the funds which were so sorely needed. Of his own knowledge amongst the dockers he could speak of the reliance placed in her by those amongst whom she laboured, and of the comfort carried into the homes of the poor in their time of need by the ministrations of the nurses of the Maternity Charity.

### THE ROYAL EYE HOSPITAL, SOUTHWARK.

The Royal Eye Hospital, or to give its more imposing title, the Royal South London Ophthalmic Hospital, is sadly in need of funds, and very special efforts are being made by those interested in its work just now to enable the whole hospital to be opened for the reception of patients. At present 18 beds are empty for want of money, and many most essential finishing touches to the fittings and arrangements have to be held over for the same reason. On Friday last the Lord Mayor, accompanied by the Lady Mayoress, presided at the festival dinner, which was held at the Grafton Galleries, Grafton Street, where the Exhibition of "Fair Women" is now open. The dinner was well attended, and at its conclusion subscriptions and donations amounting to a little over £1,000 were announced. Amongst others present were Cardinal Vaughan, Sir R. Wyatt, Sir David and Lady Evans, Sir Dixon Hartland, M.P., and Lady Hartland, Professor McHardy, and Dr. W. H. Dickenson.

In proposing the toast of the evening, the Lord Mayor made an urgent appeal for support, according his warm admiration to those good friends of the Institution, Mr. Brindley, Sir Richard Wyatt, Professor McHardy and others, who had done so much for it. The Hospital itself he and the Lady Mayoress had visited recently, and could only say they found efficiency and economy everywhere; the House Surgeon and Matron were untiring in their desire and efforts to interest people in the work of the Institution. The need for this Hospital was shown by the fact that since its establishment in 1857, 165,700 patients had been relieved within its walls. The new buildings which were opened in 1892 were now completed at a total cost of £34,000, but there was a deficiency both on the annual expenditure and the building fund, and more money was urgently needed. With the toast of "Prosperity to the Royal Eye Hospital" he would couple the name of Professor McHardy, to whom it owed so much.

Professor McHardy responded to the toast, and spoke of the keen interest he had taken in the Hospital, saying he had travelled from St. Petersburgh to Chicago in search of faults to be avoided in a building which should be a model of what hospitals ought to be. He wished especially to mention the name of Mr. Keith D. Young, the able architect of the new buildings in this connexion. He strongly urged the claims of the Royal Eye Hospital, pointing out that it is the only institution in the large district of South London for the special treatment of eye diseases. No better site could be found for such a building than the one which, by the generosity of Sir Temple West and the City of London, it now occupies. Professor McHardy went on to say that the funds of the Royal Eye Hospital were devoted wholly and solely to the benefit of the patients, not one farthing being spent on advertisements. Other toasts followed, the "Staff of the Institution" being proposed by Sir David Evans, and responded to by Dr. Spencer Watson. The speeches were necessarily very brief, as after the dinner the Lady Mayoress held a reception in the picture galleries at which many guests were present, for whose amusement various artistes kindly gave their services. The programme included songs by Miss Ethel Winn, Miss Llewella Davies, Mr. Charles Capper, Mr. Rutland Barrington, and the Dilettante Vocal Quartette. The mandoline guitar players, stationed at the far end of the galleries, proved a special attraction. The rooms were well filled all the evening, too full indeed to allow the pictured beauties on the walls their fair meed of attention. Miss Islip, the energetic Matron of the Royal Eye Hospital, was present with one or two of her nurses in pretty blue-and-white uniforms, busily dispensing programmes and a wonderfully got-up book, specially designed for the occasion (its cost being defrayed by the advertisers therein), its last page showing a pathetically empty ward "closed for want of funds." By way of additional attraction, "Palmistry, or Character Reading" was undertaken by Mr. Luther Munday, "for the benefit of the Hospital." Altogether the evening's entertainment should have resulted in substantial help for an institution which has admittedly so many claims on a charitable public. A further effort is to be made in a few weeks in the form of a concert, for which Miss Ellen Terry, Miss Marian Mackenzie, and others have kindly promised their help.

